# Diosmin: A promising phytochemical for functional foods, nutraceuticals and cancer therapy

**DOI:** 10.1002/fsn3.4271

**Published:** 2024-06-18

**Authors:** Lubna Rahman, Ali Talha Khalil, Syed Ahsan Shahid, Zabta Khan Shinwari, Zainab M. Almarhoon, Amnah Alalmaie, Javad Sharifi‐Rad, Daniela Calina

**Affiliations:** ^1^ Department of Biotechnology Quaid‐i‐Azam University Islamabad Pakistan; ^2^ Department of Pathology Lady Reading Hospital Medical Teaching Institution Peshawar Pakistan; ^3^ Department of Plant Sciences Quaid‐i‐Azam University Islamabad Pakistan; ^4^ Department of Chemistry College of Science, King Saud University Riyadh Saudi Arabia; ^5^ Department of Pharmaceutics College of Pharmacy, King Khalid University Abha Saudi Arabia; ^6^ Department of Biomedical Sciences College of Medicine, Korea University Seoul Republic of Korea; ^7^ Department of Clinical Pharmacy University of Medicine and Pharmacy of Craiova Craiova Romania

**Keywords:** anticancer properties; apoptosis, chemotherapy adjunct, diosmin, flavonoids, pharmacological applications, phytochemicals

## Abstract

Diosmin, a potent bioflavonoid derived from citrus fruits, has gained significant attention for its anticancer potential, reflecting a critical need in the ongoing battle against cancer. Amidst increasing cancer incidence, the quest for safer and more effective treatments has brought diosmin to the forefront, given its unique pharmacological profile distinct from other flavonoids. Diosmin's anticancer mechanisms are multifaceted, involving apoptosis induction, angiogenesis inhibition, and metastasis prevention. Extensive research encompassing cellular studies, animal models, and limited clinical trials underscores its efficacy not only against cancer but also in managing chronic venous insufficiency and hemorrhoids, attributing to its anti‐inflammatory properties. Furthermore, diosmin exhibits low toxicity and complements conventional chemotherapy, proposing its utility as an adjunct therapy in cancer treatment protocols. The review delves into the specific anticancer advantages of diosmin, distinguishing it from the broader flavonoid category. It provides a detailed analysis of its implications in preclinical and clinical settings, advocating for its consideration in the oncological therapeutic arsenal. By juxtaposing diosmin with other herbal medicines, the review offers a nuanced perspective on its role within the wider context of natural anticancer agents, emphasizing the need for further clinical research to substantiate its efficacy and safety in oncology.

## Graphical abstract

The anticancer mechanisms of diosmin involve stimulating receptors, increasing ROS, and inducing cell death via the PI3K/Akt pathway. Diosmin upregulates p53 and p21, causing S‐phase cell cycle arrest, and activates caspase‐3, leading to apoptosis. Additionally, diosmin inhibits NF‐κB, reducing inflammation and angiogenesis, and promotes autophagy.

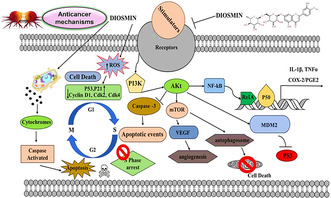

## 1 INTRODUCTION

Cancer, characterized by the uncontrolled growth and spread of abnormal cells, is a major global health concern (da Cunha et al., 2023; “Global, regional, and national burden of colorectal cancer and its risk factors, 1990–2019: a systematic analysis for the Global Burden of Disease Study 2019,” 2022). The persistent global effort to combat cancer, a major cause of mortality, has heightened the search for therapeutic compounds that can effectively and safely target and treat this complex disease (Debela et al., 2021). Medicinal plants are considered a rich source of various bioactive secondary metabolites with potential anticancer applications (Sati et al., 2024; WHO, 2022). Bioactive natural compounds derived from plants are promising and potent novel chemotherapeutic agents for cancer therapy (Atanasov et al., 2021). While these compounds, including polyphenols, are generally considered safe and beneficial at moderate doses due to their antioxidant and anti‐inflammatory properties, high doses can lead to toxicity and adverse effects. For instance, green tea polyphenols (GTPs), despite their potent antioxidative activities, have been reported to deteriorate colitis and fail to prevent colon carcinogenesis in inflamed colons when administered at high doses (0.5 and 1%) in dietary forms (Wu et al., 2023). Therefore, it is fundamental to evaluate the safety profile of these natural compounds at varying dosages (Duda‐Chodak & Tarko, 2023). Bioactive compounds are more effective with low concentration and lower cost as compared to synthetic drugs; even natural compounds at high doses showed no harmful effect and were well tolerated by patients with no harmful side effects (Huang et al., 2021; Rejhová et al., 2018). For example, Vinblastine, Vincristine, and Taxol were among the first plant‐derived chemotherapeutic agents isolated from plants like *Catharanthus roseus* and *Taxus baccata* (Sadeghi‐Aliabadi et al., 2015); similarly, Artemisinin from *Artemisia annua*, which is now used successfully for various therapies, resulted in the Nobel Prize (Efferth et al., 2015). Among different phytochemicals isolated from plants, flavonoids have a significant range of pharmacological and biochemical applications; flavonoids are isolated as secondary metabolites from many higher plants and belong to the group of natural polyphenols having a low molecular weight (Asgharian et al., 2022; Kilit et al., 2021). Flavonoids, a large family of polyphenolic compounds, include subclasses such as flavanols, flavones, isoflavones, and flavanones. These compounds have shown significant antiproliferative properties and can induce apoptosis in human carcinoma cells (Devika et al., 2022; Sharma et al., 2022). In recent years, the anticancer potential of diosmin has garnered significant attention due to its ability to modulate various cellular pathways, including those involved in apoptosis, angiogenesis, and metastasis, which positions it as a promising candidate in cancer therapy. Diosmin is a naturally occurring flavonoid glycoside commonly found in certain fruits and plants. It is predominantly found in the pericarp and pulp of citrus fruits such as oranges (*Citrus sinensis*), lemons (*Citrus limon*), and related species; these fruits are the primary commercial sources of diosmin due to their high content and easy availability (Peng et al., 2021). The tubers of *Dioscorea zingiberensis* are significant sources of diosmin; these plants are traditionally used in herbal medicine and are rich in steroidal saponins, which can be hydrolyzed to produce diosmin (Wang et al., 2023; Zhu et al., 2010). Diosmin can also be extracted from fenugreek seeds and other parts of the plant (Chaudhary et al., 2018). By‐products from the essential oil industry, such as distilled solid residues from rosemary, Greek sage, and spearmint, are also explored for diosmin extraction (Christaki et al., 2022). These residues are rich in bioactive compounds and offer a sustainable source for diosmin production (Christaki et al., 2022).

Citrus fruits have been reported for their anticancer properties, because of the existence of flavonoids in their secondary metabolites; among all these reported flavonoids, diosmin is one of them having anticancer properties. In recent years, the anticancer potential of diosmin has garnered significant attention due to its ability to modulate various cellular pathways, including those involved in apoptosis, angiogenesis, and metastasis, which positions it as a promising candidate in cancer therapy. Preliminary studies have shown encouraging results in the inhibition of tumor growth and progression in various cancer cell lines, suggesting a potential role for diosmin as an adjunct in oncology treatment regimens (Yao et al., 2021). Diosmin has been recognized for its vascular protective properties, particularly in the management of conditions such as lymphedema, varicose veins, and chronic venous insufficiency; its mechanisms of action in these contexts have been well studied (Bogucka‐Kocka et al., 2013), including its role in modulating venous tone, lymphatic drainage, and capillary permeability (Huwait & Mobashir, 2022; Patel et al., 2013; Pushkaran et al., 2019). Beyond these vascular effects, diosmin has demonstrated a broad spectrum of therapeutic benefits, ranging from anti‐inflammatory and antimicrobial activities to potent antioxidant properties; these attributes contribute to its therapeutic efficacy in a variety of oxidative stress‐related disorders (Huwait & Mobashir, 2022). The anti‐inflammatory properties of diosmin also warrant attention, particularly in the context of chronic diseases where inflammation plays a key role; its impact on inflammatory biomarkers and immune cells suggests potential benefits in conditions such as asthma, arthritis, and autoimmune diseases (Gerges et al., 2022; Pari & Srinivasan, 2010; Yao et al., 2021). Moreover, diosmin's implications in metabolic disorders, including its anti‐hyperglycemic effects in diabetes, add another dimension to its therapeutic profile (Gerges et al., 2022; Huwait & Mobashir, 2022; Kilit et al., 2021). The focus of this review is to summarize the recent studies on diosmin, with a special emphasis on its properties as an anticancer agent. The objective is to present a comprehensive understanding of the latest findings related to diosmin, including its modes of action, effectiveness in various health conditions, and capacity as a versatile therapeutic compound. This detailed examination aims to highlight the growing importance of diosmin in cancer treatment while also exploring its wider impact in the field of pharmacology. This updated review integrates the most recent studies to provide an up‐to‐date perspective on diosmin's anticancer potential and other biomedical applications.

## 2 REVIEW METHODOLOGY

A comprehensive literature search was conducted across multiple databases, including PubMed, Scopus, Web of Science, ScienceDirect, and Google Scholar, to ensure broad coverage of relevant studies (Lo, 2022). The review spans publications from 1992 to 2023, providing an extensive and up‐to‐date examination of the topic. The search strategy employed a combination of keywords and MeSH (Medical Subject Headings) terms using Boolean operators to refine the results. The keywords used were: “Diosmin” [MeSH Terms] AND (“anticancer” OR “antitumor” OR “oncology”) AND (“therapeutic applications” OR “pharmacology” OR “treatment”).

Articles were selected based on the following inclusion criteria:

- Studies that specifically focused on the anticancer properties of diosmin or its role in other therapeutic areas.
- Articles that provided clinical or preclinical data.
- Studies published in peer‐reviewed journals.
- Articles available in English.

The exclusion criteria were as follows:

- Studies not directly related to Diosmin's therapeutic applications.
- Non‐peer‐reviewed articles, conference abstracts, and book chapters.
- Studies published in languages other than English.
- Duplicate studies or studies with overlapping data.

Following the search, articles were first screened based on their titles and abstracts. Relevant articles were then subjected to a full‐text review. Data extracted from the selected studies included the study design, sample size, methodology, outcomes measured, and conclusions. This information was tabulated and analyzed to identify common themes, outcomes, and discrepancies in the research. The extracted data were synthesized narratively, focusing on the anticancer potential of diosmin and its other therapeutic applications. This synthesis involved discussing the mechanisms of action, efficacy, safety profile, and comparison with other treatments, where relevant. The findings were discussed in the context of the current landscape of cancer treatment and Diosmin's role within it. The implications for future research, clinical practice, and potential therapeutic applications were also addressed. The review concluded with a summary of key findings, the limitations of the current literature, and suggestions for future research directions in the field of diosmin's therapeutic applications. The chemical structure has been validated with PubChem (PubChem) and the taxonomy of the plants according to World Flora Online (WFO, 2023).

## 3 DIOSMIN: GENERAL CHARACTERIZATION

### 3.1 Chemical data

**FIGURE 1:**
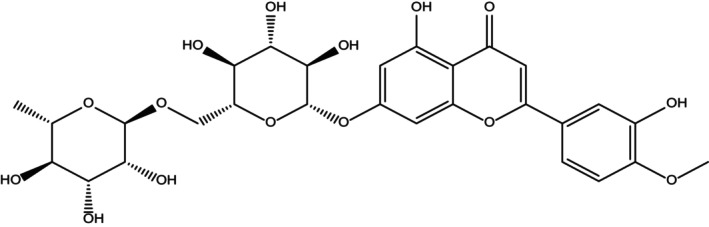
Chemical **s**tructure of diosmin.

Diosmin (3′,5,7‐trihydroxy‐4′‐methoxyflavone 7‐rutinoside) (Figure 1), with the IUPAC name [5‐hydroxy‐2‐(3‐hydroxy‐4‐methoxyphenyl)‐7‐[(2S,3R,4S,5S,6R)‐3,4,5‐trihydroxy‐6 [[(2R,3R,4R, 5R, 6S)‐3,4,5‐trihydroxy‐6‐methyloxan‐2‐yl] oxymethyl] oxan‐2‐yl] oxychromen‐4‐one] and hesperidin, whose flavanone analog belongs to the family Rutaceae, and is mostly found in citrus fruits, are common flavone glycosides (Bogucka‐Kocka et al., 2013). Diosmin was first isolated in 1925 from *Scrophularia nodosa* and introduced as a therapeutic agent for the first time in 1969 (Gervasi et al., 2023). Diosmin differs from hesperidin as it contains a double bond in its central carbon ring.

### 3.2 Bioavailability and pharmacokinetics

Diosmin is mainly utilized for treating venous and lymphatic disorders; however, recent evidence indicates its potential therapeutic effects in other areas, such as cancer treatment (Mustafa et al., 2022). The bioavailability of diosmin refers to the degree and speed at which the drug is absorbed into the bloodstream and available for use by the body (Ai et al., 2014). Nevertheless, oral administration results in rapid absorption from the gastrointestinal tract, with peak plasma concentrations occurring within 2–4 hours (Bogucka‐Kocka et al., 2013). The absorption of diosmin is affected by several factors, such as its formulation, dosage, and whether it is taken with food. The presence of food in the stomach and by physical modifications techniques like media milling/nanocrystal technology, cryogenic technology, supercritical fluid process, modification of the crystal habit, complexation, micellar technologies, chemical modifications, and other techniques like co‐crystallization, co‐solvency, and hydrotrophy have been shown to increase diosmin bioavailability by up to 50%, enhancing its solubility and absorption (Abd El Hady et al., 2019; Chaudhary et al., 2012).

Diosmin exhibits poor water solubility, which significantly limits its bioavailability. Various strategies have been explored to enhance its solubility. One effective method involves the use of cyclodextrin complexation. Inclusion complexes with β‐cyclodextrin (β‐CD) and hydroxypropyl‐β‐cyclodextrin (HPβCD) significantly improve the solubility and dissolution rate of diosmin. Phase solubility studies have confirmed a 1:1 molar ratio for these complexes, resulting in improved stability and bioactivity. The addition of hydroxypropyl methyl cellulose (HPMC) and polyethylene glycol 6000 (PEG 6000) to β‐cyclodextrin complexes further increases diosmin's solubility and in vitro bioactivity (Anwer & Shakeel, 2015) (Ai et al., 2014)The solubility of diosmin was found to be significantly enhanced in β‐cyclodextrin aqueous solutions and polyethylene glycol‐400 (PEG‐400) compared to water alone (Anwer & Shakeel, 2015).

The pharmacokinetics of diosmin refers to the study of the time course of drug absorption, distribution, metabolism, and excretion in the body. Diosmin has a relatively long half‐life and a low clearance rate, allowing for sustained drug levels and prolonged therapeutic effects (Cova et al., 1992). Studies on the half‐life of diosmin in animals have shown that diosmin, when complexed with phospholipids, has an extended elimination half‐life, which enhances its bioavailability and retention time in the body (Adouani et al., 2020). In another pharmacokinetic study in healthy volunteers, the aglycone form of diosmin, diosmetin, demonstrated a long plasma elimination half‐life ranging from 26 to 43 hours, indicating significant metabolism prior to absorption (Cova et al., 1992).

The bioavailability of diosmin is estimated at approximately 55% absorption by the body, with more than 55% of the administered dose available for use by the body (Russo et al., 2018). Diosmin is extensively distributed in the body with a high volume distribution, and it accumulates in the endothelial cells of the veins and lymphatic vessels, where it exerts its pharmacological effects (Sarhan et al., 2021). Diosmin does not bind extensively to plasma proteins, which contributes to its widespread distribution in the body. In the liver, diosmin undergoes extensive first‐pass metabolism, where it is converted into its active metabolite, diosmetin, which is a flavonoid aglycone responsible for most of the pharmacological effects of diosmin (Benavente‐Garcia & Castillo, 2008; Gerges et al., 2022). Diosmetin is further metabolized into various metabolites, which are excreted in the urine and feces (Chen, Xu, et al., 2019). The elimination half‐life of diosmin varies depending on the administered dose; the elimination half‐life depends on the dose ingested, but its action duration does not have a direct relationship with the plasmatic half‐life because of the bio‐transformation pathways (Virseda‐Rodríguez et al., 2015). Diosmin is primarily eliminated via the feces, with only a small fraction excreted in the urine (Chen, Xu, et al., 2019). Evidence indicates that diosmin has potential anti‐cancer effects, particularly in treating colorectal cancer (Tanaka, Makita, Kawabata, et al., 1997). In preclinical studies, diosmin has been shown to inhibit the growth and proliferation of cancer cells, induce apoptosis, and inhibit angiogenesis (Huwait & Mobashir, 2022). Colorectal cancer is the third most common cancer globally and is often associated with a poor prognosis. In vitro studies have shown that diosmin inhibits the growth and proliferation of colorectal cancer cells, inducing cell cycle arrest and apoptosis (Zeya et al., 2022). In animal models of colorectal cancer, diosmin has been shown to inhibit tumor growth and reduce tumor size (Helmy et al., 2020). Apart from its anti‐cancer effects, diosmin has been studied for its potential therapeutic effects in several other conditions. According to recent pharmacokinetic studies, diosmin is rapidly hydrolyzed to its aglycone form, diosmetin, by intestinal microflora enzymes, which is easily absorbed and dispersed throughout the body. It is also broken down to glycin‐conjugated derivatives or phenolic acids, Intestinal flora rapidly transformed diosmin to its aglycon form, diosmetin, according to pharmacokinetic investigations, which is absorbed and distributed rapidly with a plasma half‐life of 26–43 hours throughout the body. Diosmetin is converted to its glycine‐conjugated derivatives or phenolic acids and eliminated from the urine (Gerges et al., 2022). Various metabolic enzymes' inhibitory applications of diosmin help to investigate its promising therapeutic efficacy and safety in various types of diseases (Abdel‐Reheim et al., 2017; Gerges et al., 2022; Waring et al., 2015).

## 4 CHEMOPREVENTIVE AND ANTICANCER ACTIVITY OF DIOSMIN

### 4.1 Chemopreventive effect

Chemoprevention, a proactive strategy in cancer management, involves the use of natural or synthetic agents to thwart the development or progression of cancer, thereby providing a vital approach to reducing the global burden of this disease (G et al., 2022). In a recent study investigating bladder cancer prevention, diosmin and hesperidin, two flavonoids, were found to significantly reduce bladder carcinoma and preneoplasia in mice (Yang et al., 1997). The study involved administering these compounds, either individually or in combination, to male ICR mice exposed to the carcinogen OH‐BBN. This treatment was shown to lower the incidence of bladder lesions; importantly, the research also indicated that these flavonoids suppress cell proliferation, as evidenced by reduced counts of silver‐stained nucleolar‐organizer‐region‐associated proteins (AgNORs) and lower 5‐bromodeoxyuridine (BUdR)‐labeling indices in bladder lesions (Yang et al., 1997). These findings suggest the potential of diosmin and hesperidin to inhibit bladder cancer through the suppression of cellular proliferation (Yang et al., 1997). Diosmin in Nitrosodiethylamine (NDEA) hepatocarcinogenesis induced in rats showed a chemopreventive effect through restoring the alteration of Mcl‐1, Bax, Bad, AFP, Bcl‐2, Bcl‐xL, CAT, GPx, GSH, VitE, VitC, SOD, caspase‐3, 9 and lipid peroxidation (Perumal et al., 2018). In a recent study on an experimental mouse liver cancer model, diosmin decreased the growth of HA22T cells in human hepatocellular cancer by downregulating the PI3K–Akt–MDM2 signaling pathway and triggering G2/M cell cycle arrest (Dung et al., 2012). Another recent study showed that diosmin effectively prevents the progression and development of carcinomas and triggers caspase‐dependent apoptosis in the DMBA‐induced hamster buccal pouch (HBP) (Rajasekar et al., 2016). Similarly, diosmin in acute myeloid leukemia treatment caused a decrease in tumor cell growth through caspase‐8 activation and increased TNF‐α expression (Roma et al., 2018) and also downregulated the active proteins in the G0–G1 cell cycle phase, like Cdk2, Cdk4, and cyclin D1. Diosmin also decreases the production of c‐Myc and Bcl‐2 genes while elevating the production of FOXO3a, p27Kip1, and Bax (Oak et al., 2018).

### 4.2 Anticancer mechanisms

#### 4.2.1 Apoptosis induction

Apoptosis, a regulated process of programmed cell death, plays a critical role in cancer biology, both in the suppression of tumor initiation and the effectiveness of cancer therapies, by selectively eliminating damaged or abnormal cells (Pfeffer & Singh, 2018; Singh et al., 2022). Many studies showed that diosmin has pro‐apoptotic dose‐dependent effects on several cancers, like prostate, oral, colon, urinary bladder, and breast tumors. In recent studies, diosmin increased intracellular redox disequilibrium or oxidative stress in tumor cells, which caused changes in the potential of the mitochondrial membrane and led to apoptotic cell death. It also caused tumor cell death by forming micronuclei (genotoxicity) and introducing double‐stranded breaks in the DNA (Lewinska et al., 2017). In breast cancer cell lines MDA‐MB‐231, SK‐BR‐3, and MCF‐7, diosmin exhibits apoptosis through different responses, including: G2/M cell cycle arrest; ERK1/2 activation; premature senescence; p53, p27, and p21 level elevation; increased SA‐βgal activity; DNA damage; increasing the oxidative stress in tumor cells; and autophagy (Lewinska et al., 2017). In the case of liver cancer cells HepG2 and HCC‐LM3, the diosmin in their aglycon form, diosmetin, caused apoptosis due to proliferation suppression and by arresting the G2/M phase of the cell cycle (Ma & Zhang, 2020). Diosmin increases p53, p21, and p27 levels; induces G2/M cell cycle arrest; boosts SA‐β‐gal (senescence‐associated beta‐galactosidase) activity and oxidative stress; and controls DNA damage (Anna Lewinska et al., 2017). Diosmin caused the elevation of p21, p53, and p27 levels in the cell, which led to the G2/M cell cycle arrest, also increased oxidative stress, DNA damage, and SA‐βgal activity in MCF‐7 cells and caused the aging of the cell. Recent studies showed that diosmin‐induced apoptosis in renal carcinoma ACHN cells by upregulating p53 expression, PI3K/AKT, and targeting Chk2 in HepG2 cancer cells caused the apoptosis (Serra et al., 2021). Diosmin, through a ROS‐mediated mechanism, overexpression of caspases 9 and 3, p53, and downregulation of Bcl‐2 and MMP‐2,9 (matrix metalloproteinases 2 and 9) caused apoptosis in skin cancer cells (A431) (Paredes et al., 2018). In the case of breast cancer, the MDA‐MB‐231 and SKBR‐3 cell lines didn't show a response to diosmin, while in the case of the MCF‐7 cell line, at various doses, it caused premature senescence and apoptosis. The increased levels of reactive oxygen species (ROS), mitochondrial production, and total superoxide, along with heightened protein carbonylation and nitric acid, have been correlated with the triggering of apoptosis in cancer cells (Lewinska et al., 2017; Perumal et al., 2018). Diosmin causes the apoptosis of cancer cells by increasing the levels of total reactive oxygen species (ROS), total superoxide, nitric oxide, protein carbonylation, and mitochondrial production, which are also determined by the pro‐apoptotic activity of diosmin on the DU145 androgen‐independent prostate cancer cell line. (Lewinska et al., 2015). In another recent study, diosmin was reported to have pro‐apoptotic activity, like in the androgen‐independent prostate cancer cell line (DU145), through intracellular oxidative stress, redox disequilibrium, an overall boost in ROS production, increase the number of micronuclei (genotoxicity), and DNA double‐stranded break formation (Chikara et al., 2018). Diosmin showed anticancer activity in hepatocarcinoma, breast, and colorectal cancer cells with the activation of Fas and Bax apoptotic factors, which cause the apoptosis of the cell also through cyt C (cytochrome C) releasing from mitochondria, inhibiting the translocation of NF‐кB, activating different caspases, and triggering the cell cycle in the G2/M phase (Koosha et al., 2019). Diosmin, through a ROS‐mediated mechanism, overexpression of caspase 9 and 3, p53 genes, and downregulation of Bcl‐2 and matrix metalloproteinases‐2 and 9 genes caused apoptosis in skin cancer cells (A431) (Buddhan & Manoharan, 2017). Similarly, diosmin in GBM02 and GBM95 glioblastoma cells induces caspase‐dependent apoptosis (Soares et al., 2019). In the case of DU145 cells, diosmin, by increasing the production of ROS, micronuclei, and DNA double‐strand breaks, exhibits genotoxicity and stimulates intracellular redox disequilibrium (Lewinska et al., 2017). Diosmin also causes the inhibition of rate‐limiting enzymes. In colonic cancer treatments such as ornithine decarboxylase (ODC), inhibition of ODC due to DNA damage causes cell apoptosis, which affects the growth of the tumor (Huwait & Mobashir, 2022). Diosmin also used dose‐dependent activity for A431 skin cancer cells, by inducing apoptosis through overexpression of caspase 3, p53, and caspase 9 gene overexpression DNA fragmentation, which caused the downregulation of MMP‐2, 9 (matrix metalloproteinases‐2, 9) and Bcl‐2 genes and also ROS‐mediated mechanisms (Eraslan et al., 2017; Perumal et al., 2018). Also, it induced apoptosis of acute myeloid leukemia cells by interaction with estrogen receptor‐β (ERβ) and triggered TNF‐α mediated extrinsic apoptotic pathway (Roma et al., 2018; Roma & Spagnuolo, 2020). In the case of colorectal cancer cell lines (HCT‐116), diosmin caused apoptosis by inactivating NF‐кB translocation, releasing from mitochondria the cytochrome C, and arising caspases and apoptotic factors Fas and Bax (Koosha et al., 2019). Diosmin has also been reported to cause apoptosis in DU145 prostate cancer cells, SKBR‐3, MDA‐MB‐231, and MCF‐F breast cancer cells, and in the skin cancer cell (A431) (Lewinska et al., 2017; Yao et al., 2021). MCF‐7 cells were found to be the most responsive to the compound diosmin in the case of breast cancer as compared to the others. At lower doses, it caused the G2/M cell cycle arrest and elevated p2, p27 and p53 levels, causing oxidative stress, DNA damage, and increased SA‐βgal activity, all of which are associated with the aging of cancer cells. Diosmin also led to an increased level of nitric oxide, total superoxide, protein carbonylation, total ROS (reactive oxygen species), and mitochondrial production, which caused the apoptosis of the cancer cells.

#### 4.2.2 Inhibition of angiogenesis and anti‐proliferative effects

The synergistic relationship between the inhibition of angiogenesis and anti‐proliferative effects is pivotal in cancer treatment, as it disrupts the tumor's blood supply while simultaneously curbing the uncontrolled proliferation of cancer cells, effectively impeding tumor growth and spread (Zuazo‐Gaztelu & Casanovas, 2018). Recent studies showed that diosmin inhibited tumor cell proliferation by normalizing tumor vasculature, decreasing angiogenesis and accelerating caspase pathway (Choi et al., 2019). Diosmin has been included as a promising anti‐proliferative agent against 2‐AAF and DEN‐stimulated hepatocarcinogenesis and hyperproliferation in rats, where diosmin decreases the production of serum cytotoxicity factors (ALT, LDH and AST), oxidative stress, promotion factors of tumors (PCNA, Ki67, and ODC), pro‐inflammatory cytokines (iNOS and COC‐2), cytotoxicity factors of serum (ALT, LDH, AST), and necrosis factors of cells (TNFα, NF‐κB) (Tahir, Rehman, Lateef, Khan, Khan, Qamar, O'Hamiza, et al., 2013a). According to a recent study, diosmin showed dose‐dependent anti‐proliferative activity for various types of cancers, like prostate, oral, breast, urinary bladder and colon. In human hepatocellular carcinoma, HA22T cells treated with diosmin showed promising inhibitory effects on viability and proliferation by modulating PI3K‐AKT/MDM2 signaling pathways and p53, the downstream factors of the phosphate 2A (PP2A) protein (Dung et al., 2012). Diosmin was also found to inhibit the activity of the signal transducer and activator of transcription 3 (STAT3) signaling pathway, which is involved in tumor growth and survival (Lewinska et al., 2017). These findings suggest that diosmin may be able to inhibit key signaling pathways involved in cancer development and progression, potentially reducing tumor growth and improving patient outcomes.

#### 4.2.3 Antiangiogenic and antimetastatic effects

**FIGURE 2:**
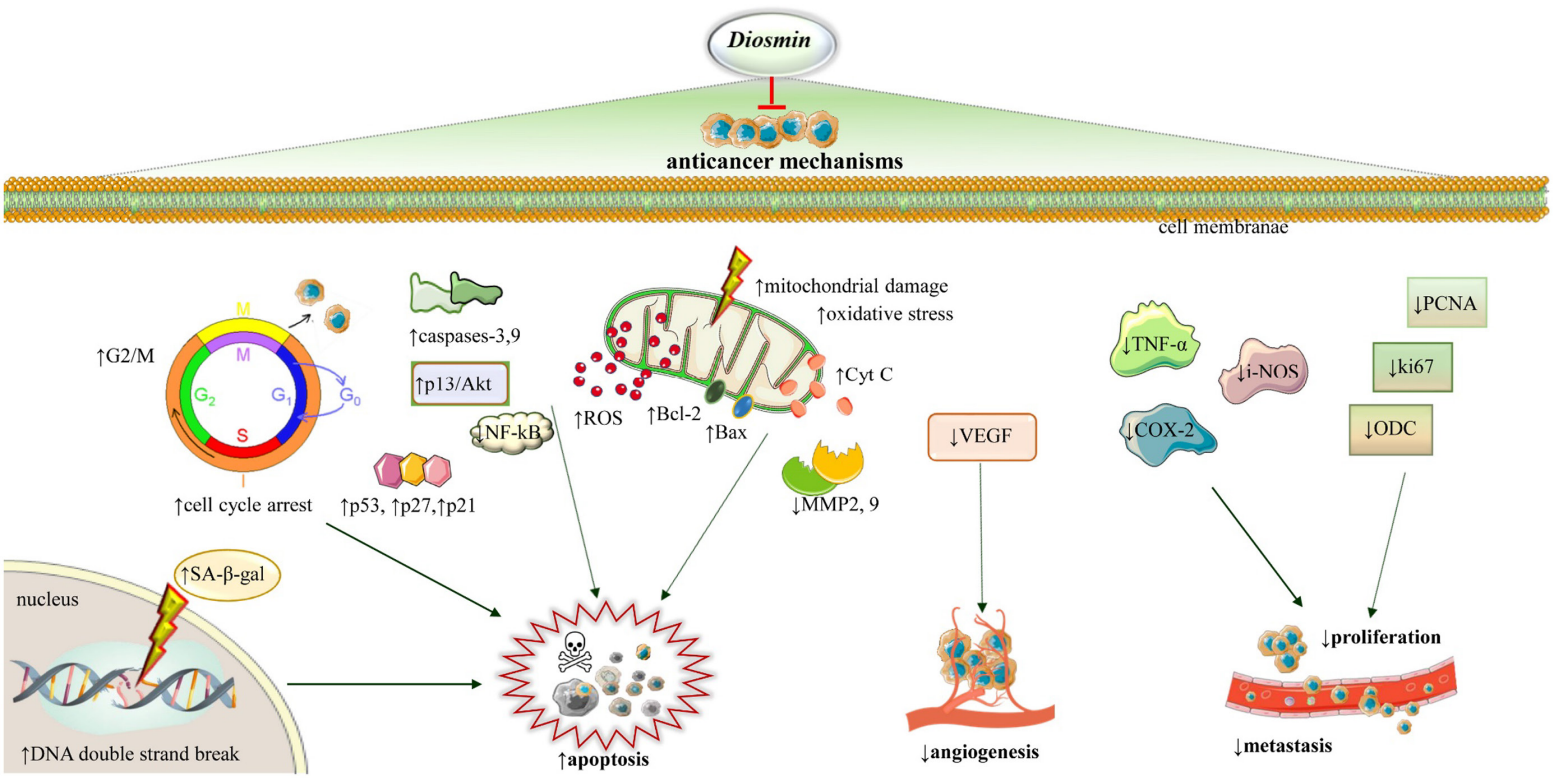
Multifaceted anticancer effects of diosmin: molecular pathways and mechanisms. Diosmin induces cell cycle arrest by upregulating tumor suppressors like p53, p27, and p21, particularly at the G2/M checkpoint, and promotes DNA damage through increased double‐strand breaks. It triggers apoptosis via caspase activation, mitochondrial damage, and ROS generation, leading to the release of cytochrome c. The compound also impedes angiogenesis by reducing VEGF levels and hinders metastasis by lowering MMP2 and MMP9 expression. Additionally, diosmin modulates inflammatory pathways by downregulating TNF‐α and COX‐2, along with i‐NOS, thereby attenuating tumor proliferation and inflammation. It decreases the proliferation markers PCNA and Ki67 and induces cellular senescence, as evidenced by elevated SA‐β‐gal activity. Akt, protein kinase B; Bax, Bcl‐2‐associated X protein; Bcl‐2, B‐cell lymphoma 2; Cyt C, cytochrome C; COX‐2, cyclooxygenase‐2; DNA, deoxyribonucleic acid; G0/G1/S/G2/M, phases of the cell cycle; i‐NOS, inducible nitric oxide synthase; Ki67, a marker of proliferation; MMP2, 9, matrix metalloproteinase 2 and 9; NF‐kB, nuclear factor kappa‐light‐chain‐enhancer of activated B cells; PCNA, proliferating cell nuclear antigen; ROS, reactive oxygen species; SA‐β‐gal, senescence‐associated beta‐galactosidase; TNF‐α, tumor necrosis factor alpha; VEGF, vascular endothelial growth factor. Symbols: ↑ increase/upregulation; ↓ decrease/downregulation.

Angiogenesis is a critical step in tumor growth and metastasis (Liu et al., 2023), and diosmin has also been shown to inhibit angiogenesis, which is the formation of new blood vessels that supply tumors with nutrients. In a recent study diosmin was shown to inhibit the expression of vascular endothelial growth factor (VEGF), which is a key regulator of angiogenesis. The study found that diosmin treatment significantly decreased the levels of VEGF in the tumor microenvironment, indicating a potential anti‐angiogenic effect (Feldo et al., 2019). Diosmin has also been shown to modulate the activity of various molecules involved in angiogenesis, the formation of new blood vessels that supply tumors with nutrients. In a study, diosmin was found to inhibit the activity of vascular endothelial growth factor (VEGF), a key molecule involved in tumor angiogenesis (Helmy et al., 2020). Metastasis, the process by which cancer cells spread from the primary site to distant organs, is a critical and complex stage in the progression of cancer, posing significant challenges to effective treatment (Buga et al., 2019; Fares et al., 2020; Gerstberger et al., 2023; Zlatian et al., 2015). Diosmin was found to show antimetastatic activity in B16F10 cells by decreasing the implantation percentage (79.40%), invasion index (45.23%), metastatic nodule number (22%), and growth index (67.44%) (Martínez Conesa et al., 2005). In melanoma lung metastatic (B16F10) cancer, diosmin has been reported to have anti‐metastatic properties (Martinez et al., 2005), where the diosmin lowers the invasion index in both macrosopic and microscopic investigation, implant percentage, and the number of metastatic nodules (Alvarez et al., 2009). Diosmin also has an anticarcinogenic effect on the 4‐NQO cell by inhibiting DT‐diaphorase activity. Diosmin in cancer treatment decreases the implant percentage, invasion index, and metastatic nodules, causing the death of the cell (Kilit et al., 2021). Figure 2 summarizes the diverse mechanisms through which diosmin exerts its anticancer properties.

### 4.3 Immunomodulatory activity

The immune system plays a critical role in the defense against cancer by recognizing and eliminating cancer cells. However, cancer cells can evade the immune system by various mechanisms, such as downregulating immune checkpoint proteins and secreting immunosuppressive cytokines (Tahir, Rehman, Lateef, Khan, Khan, Qamar, Ali, et al., 2013b; Yang et al., 2019). Therefore, the development of immunomodulatory agents has become an important strategy for cancer treatment. In addition to its direct anti‐cancer effects, diosmin has also been shown to modulate the immune system in cancer patients; it possesses immunomodulatory effects that can potentially enhance the ability of the immune system to recognize and eliminate cancer cells (Huwait & Mobashir, 2022).

#### 4.3.1 Diosmin's effects on immune cells: Macrophages, lymphocytes, and T cells

One way in which diosmin modulates the immune system is by regulating the activity of immune cells such as macrophages and lymphocytes. Macrophages are immune cells that play a key role in the immune response to cancer, with some macrophages promoting tumor growth while others inhibiting it (Imam et al., 2015). Diosmin was found to selectively modulate the activity of macrophages, promoting the activation of anti‐tumor macrophages and inhibiting the activity of pro‐tumor macrophages, and recent studies have shown that diosmin may have potential therapeutic effects in cancer treatment, particularly through its immunomodulatory effects (Kuntz et al., 1999; Lichota et al., 2019). Similarly, diosmin has been shown to modulate the activity of lymphocytes, another type of immune cell that plays a critical role in cancer immunity. It was found to increase the activity of natural killer (NK) cells, a type of lymphocyte that directly kills cancer cells. Diosmin was also found to enhance the expression of molecules involved in NK cell activation and recruitment, suggesting that it may be able to enhance the anti‐tumor activity of NK cells (Mustafa et al., 2022).

Diosmin was reported to enhance the anti‐tumor immune response in a mouse model of colorectal cancer (Zeya et al., 2022). It has been observed that treatment significantly increased the infiltration of CD4+ and CD8+ T cells in the tumor microenvironment, indicating an enhancement of the anti‐tumor immune response.

#### 4.3.2 Inhibition of immunosuppressive cytokines

Diosmin treatment was also found to decrease the levels of immunosuppressive cytokines, such as interleukin‐10 (IL‐10) and transforming growth factor‐beta (TGF‐β), in the tumor microenvironment. IL‐10 and TGF‐β are known to inhibit the immune response and promote tumor growth (Arab et al., 2015).

#### 4.3.3 Impact on signaling pathways

**FIGURE 3:**
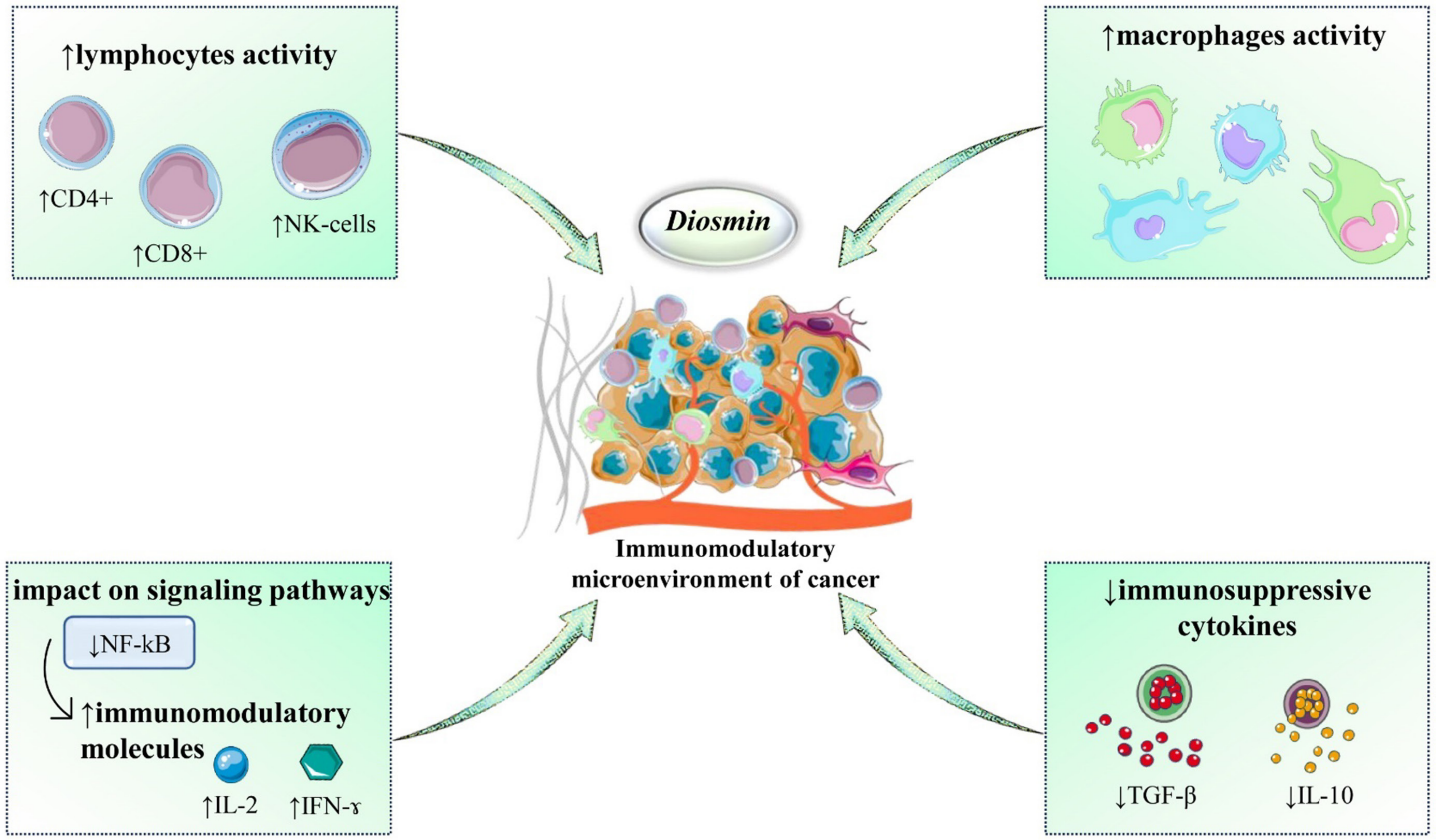
Illustrative scheme regarding diosmin‐induced immunomodulation: enhancing anticancer immune responses. Diosmin enhances lymphocyte activity, increasing the number of CD4+ and CD8+ T cells along with NK (natural killer) cells, all vital components of the adaptive and innate immune responses. It also boosts macrophage activity, which plays an important role in antigen presentation and phagocytosis. Concurrently, diosmin modulates signaling pathways by reducing NF‐kB, a key regulator of inflammation and cell survival, thereby decreasing the production of immunomodulatory molecules. This leads to an increase in the secretion of interleukin‐2 (IL‐2) and interferon‐gamma (IFN‐γ), which are pivotal for T‐cell proliferation and activation. Additionally, diosmin suppresses the release of immunosuppressive cytokines like TGF‐β and IL‐10, which are often exploited by cancer cells to evade immune detection and destruction. CD4+, cluster of differentiation 4 positive T cells; CD8+, cluster of differentiation 8 positive T cells; IFN‐γ, interferon‐gamma; IL‐2, interleukin‐2; IL‐10, interleukin‐10; NF‐kB, nuclear factor kappa B; NK cells, natural killer cells; TGF‐β, transforming growth factor beta. Symbols: ↑ increase/upregulation, ↓ decrease/downregulation.

Diosmin has also been shown to modulate the activity of various immune signaling pathways involved in cancer development and progression. In recent research, diosmin was found to inhibit the activity of the nuclear factor‐kappa B (NF‐κB) signaling pathway, which plays a critical role in cancer inflammation and progression (Huwait & Mobashir, 2022). In a recent study, it was reported that diosmin administration led to the suppression of NF‐kB activation and NF‐kB signaling. This indicates that the protective effects of diosmin may be linked to its ability to inhibit NF‐kB signaling, which is known to regulate the production of cytokines like interleukin‐2 (IL‐2) and interferon‐gamma (IFN‐γ) that are important for mounting an effective immune response against cancer. Therefore, diosmin has the potential to enhance the overall anti‐tumor immune response and, consequently, improve patient outcomes (Imam et al., 2015). Figure 3 presents the immunomodulatory effects of diosmin on the cancer microenvironment. Table 1 provides a detailed overview of the anticancer mechanisms of diosmin, illustrating its diverse effects on various cancer types.

**TABLE 1:** Anticancer mechanisms of diosmin.

Anticancer mechanisms	Cancer type	Cellular/molecular mechanism	Effects of diosmin	References
Chemopreventive	Bladder cancer (Mice)	Administering carcinogen‐exposed mice	↓ Bladder preneoplasia and carcinoma ↓ Cell proliferation	Yang et al. (1997)
	Hepatocarcinogenesis (Rats)	Restoring the alteration of various cellular factors	Prevention of NDEA‐induced carcinogenesis	Perumal et al. (2018)
	Liver cancer (Mouse model)	↓ PI3K–Akt–MDM2 ↑ G2/M cell cycle arrest	↓ HA22T cell growth	Dung et al. (2012)
	Hamster buccal pouch carcinoma	Triggering caspase‐dependent apoptosis	Prevention of carcinoma progression	Rajasekar et al. (2016)
	Acute myeloid leukemia	↑ Caspase‐8 activation; ↑ TNF‐α expression; ↓ c‐Myc, Bcl‐2; ↑FOXO3a, ↑p27Kip1, ↑Bax	↓ Tumor growth	Roma et al. (2018) Oak et al. (2018)
Apoptosis	Prostate, oral, colon, urinary bladder, breast cancer cells	↑ Redox disequilibrium ↑ Oxidative stress ↑ DNA damage	↓ ROS ↓ Mitochondrial dysfunction ↓ Micronuclei formation	Pfeffer and Singh (2018); Singh et al. (2022)
	Breast (MDA‐MB‐231, SK‐BR‐3, MCF‐7)	↑ G2/M cell cycle arrest ↑ ERK1/2 activation; ↑ p53, ↑ p27 ↑ p21↑ SA‐βgal activity ↑ Autophagy	↑ Premature senescence ↑ Oxidative stress in tumor cells	Lewinska et al. (2017)
	Liver (HepG2, HCC‐LM3)	↑ G2/M phase arrest ↓ Proliferation	↑ p21, ↑p53, ↑p27 Oxidative stress ↑ SA‐βgal activity	Ma and Zhang (2020)
	Renal carcinoma (ACHN)	↑ p53 expression ↑ PI3K/AKT modulation Targeting Chk2	↑ Apoptosis	Serra et al. (2021)
	Skin (A431)	ROS‐mediated mechanisms: ↑ Caspase 9, ‐3↓ Bcl‐2 ↓ MMP 2, ‐9	↑ Oxidative stress; ↑DNA fragmentation	Paredes et al. (2018)
	Prostate (DU145)	Intracellular oxidative stress ↑ ROS production ↑ Genotoxicity	↑ Formation of micronuclei; ↑ DNA double‐stranded breaks	Chikara et al. (2018)
	Hepatocarcinoma, breast, and colorectal cancer cells	↑ Fas, ↑ Bax ↓ NF‐кB translocation ↑ Cyt C release	↑ Cell cycle arrest in G2/M phase; ↑ Caspases activation	Koosha et al. (2019)
	Glioblastoma (GBM02, GBM95)	Caspase‐dependent mechanism	↑ Apoptosis	Soares et al. (2019)
	Acute myeloid leukemia	Interaction with ERβ; TNF‐α mediated pathway	↑ Extrinsic apoptotic pathway	Roma et al. (2018); Roma and Spagnuolo (2020)
	Colorectal (HCT‐116)	↑ NF‐кB ↑ Cyt C release from mitochondria	↑ Caspases ↑ Apoptotic factors ↑ Fas, ↑ Bax	Koosha et al. (2019)
Anti‐proliferative	Various cancers (prostate, oral, breast, etc.)	Normalizing tumor vasculature; ↓ angiogenesis; caspase pathway acceleration	↓ Tumor cell proliferation	Zuazo‐Gaztelu and Casanovas (2018) Choi et al. (2019)
	Hepatocarcinogenesis (rats)	↓ Serum cytotoxicity factors (ALT, LDH, AST); ↓Oxidative stress; ↓Tumor promotion factors: ↓ PCNA, ↓Ki67, ↓ ODC	↓ Hyperproliferation in hepatocarcinogenesis	Tahir, Rehman, Lateef, Khan, Khan, Qamar, O'Hamiza, et al. (2013a)
	Hepatocellular carcinoma (HA22T)	Modulating PI3K‐AKT/MDM2 signaling pathways; ↓ p53	↓ Cell viability ↓ Proliferation	Dung et al. (2012)
Anti‐angiogenic	Various cancer cell lines	↓ VEGF expression ↓ VEGF activity	↓ New blood vessel formation ↓ Tumor growth ↓ Metastasis	Helmy et al. (2020)
Antimetastatic	Melanoma (B16F10)	↓ Implantation percentage ↓ Invasion index ↓ Metastatic nodules	↓ Metastasis	Martínez Conesa et al. (2005) Alvarez et al. (2009)
Immunomodulation	Colorectal cancer (mouse model)	↑ CD4 +, ↑ CD8+ T cell infiltration	↑ Anti‐tumor immune response ↑Immunosuppressive cytokines: ↑ IL‐10, ↑TGF‐β	Zeya et al. (2022) Arab et al. (2015)
	Various cancers	↑ NK cells ↓ NF‐κB, ↓ STAT3	↑ NK cell activity ↓ Pathways involved in cancer inflammation and progression	Imam et al. (2015); Mustafa et al. (2022)

Abbreviations: Akt, protein kinase B; ALT, alanine Aminotransferase; AST, aspartate aminotransferase; Bax, Bcl‐2‐associated X protein; Bcl‐2, B‐cell lymphoma 2; CD, cluster of differentiation; ERK1/2, extracellular signal‐regulated kinases ½; LDH, lactate dehydrogenase; MMP, matrix metalloproteinases; NF‐κB, nuclear factor kappa‐light‐chain‐enhancer of activated B cells; NK, natural killer; ODC, ornithine decarboxylase; PCNA, proliferating cell nuclear antigen; PI3K, phosphoinositide 3‐kinases; ROS, reactive oxygen species; SA‐βgal, senescence‐associated beta‐galactosidase; STAT3, signal transducer and activator of transcription 3; TNF‐α, tumor necrosis factor alpha; VEGF, vascular endothelial growth factor. Symbols: ↑: increase/enhancement; ↓: decrease/reduction.

### 4.4 Pharmacological studies regarding the anticancer efficacy of diosmin

Numerous experimental studies have investigated the anticancer potential of diosmin, with promising results.

In vitro studies have demonstrated that diosmin can induce apoptosis (programmed cell death) in various cancer cell lines, including breast cancer, cervical cancer, liver cancer, prostate cancer, and colorectal cancer. Diosmin has been shown to activate the intrinsic apoptotic pathway, leading to the activation of caspases, which are enzymes that play a critical role in the execution of apoptosis (Dholakiya & Benzeroual, 2011; Soares et al., 2019). In addition, diosmin has been shown to inhibit cancer cell proliferation, migration, and invasion by regulating various signaling pathways, including the PI3K/Akt/mTOR pathway, the MAPK/ERK pathway, and the NF‐κB pathway. These pathways are involved in the regulation of cell survival, proliferation, and inflammation, all of which are key processes in cancer development and progression (Singh et al., 2021) (Table 2).

**TABLE 2:** Diosmin mode of action against different cancer types.

Type of cancer/tested cells lines	Model	IC_50_ (for in vitro) dose (for in vivo)	Mechanism of action	References
Glioblastoma	GBM95 and GBM02 cells In vitro	‐A	↑ Caspase‐dependent apoptosis	Soares et al. (2019)
Skin cancer	A431 cells In vitro	45 μg/mL	↑ ROS ↑ p53, ↑ caspase 3, ↑ caspase 9 ↑ DNA fragmentation	Buddhan and Manoharan (2017)
	B16F10 cells In vitro	15–60 μM	Anti‐metastatic effect by reducing the number of metastatic nodules.	Martinez et al. (2005)
Breast cancer	MCF‐7 Cells In vitro	13.93 μM	↑ Cell cycle arrest in G2/M phase ↑ Oxidative stress ↑ DNA damage	Lewinska et al. (2017)
	MDA‐MB‐231 Cells In vitro	19.46 μM	↑ p53, ↑ p21, ↑ p27, ↑ SA‐β‐gal	Lewinska et al. (2017)
	SKBR‐3 cells In vitro	17.92 μM	↑ Oxidative stress ↑ DNA damage	Lewinska et al. (2017)
Oral cancer	Rats F344 In vivo	1000 ppm	↓ ODC ↓ DT‐diaphorase activity ↑ DNA damage, ↑ cancer cell death	Browning et al. (2005) {Browning, 2005 #26}
Esophageal cancer	Wistar rats In vivo	1000 ppm	↓ Metastatic nodules, implant percentage and invasion index	Martinez et al. (2005)
Hepatocellular carcinoma	Nude mice In vivo	1000 ppm	↓ PI3K–Akt–MDM2, ↑ p53 ↑ Cell cycle arrest at G2/M phase	Dung et al. (2012)
	HepG2 cells In vitro	–	↓ PI3K/AKT ↑ p53	Helmy et al. (2020)
	HA22T cells In vitro	–	↑ p53, ↑ PI3K‐AKT/MDM2 ↓ PP2A	Dung et al. (2012)
Colon cancer	HT‐29 cells In vitro	76.5 ± 6.5 μM	↑ Oxidative stress	Mustafa et al. (2022)
	Caco‐2 cells In vitro	112.2 ± 6.9 μM	↓ P‐glycoprotein mediated efflux of drugs	Mustafa et al. (2022)
	Rats F344 In vivo	1000 ppm	↓ ODC ↑ DNA damage	Huwait and Mobashir (2022)
	HCT‐116 In vitro	–	↓ Proliferation ↑ Apoptosis ↓ PI3K/AKT/mTOR/NF‐κB	Helmy et al. (2020)
Renal carcinoma	ACHN cells In vitro	–	↓ Cellular proliferation ↑ Apoptosis by targeting Chk2	Ma and Zhang (2020)
Urinary‐bladder cancer	ICR Mice In vivo	1000 ppm	↓ Cell proliferation	Yang et al. (1997)
Prostate cancer	DU145 cells In vitro	150 μM	↑ Oxidative stress ↑ Apoptotic cell death	Lewinska et al. (2015)

Abbreviations: ACHN cells, human renal carcinoma cell line; B16F10 cells, murine melanoma cell line; Caco‐2 cells, human colorectal adenocarcinoma cell line; DU145 cells, human prostate cancer cell line; GBM02 cells, human glioblastoma cell line; GBM95 cells, human glioblastoma cell line; HA22T cells, human hepatocellular carcinoma cell line; HepG2 cells, human liver cancer cell line; HT‐29 cells, human colorectal adenocarcinoma cell line; HCT‐116, human colorectal carcinoma cell line; IC_50_, half maximal inhibitory concentration; ICR Mice, strain of mice used in research; MCF‐7 Cells, human breast cancer cell line; MDA‐MB‐231 Cells, human breast cancer cell line; Nude mice, strain of mice used in medical research; ODC, ornithine Decarboxylase; PI3K, phosphoinositide 3‐kinase; ppm, parts per million; Rats F344, strain of rats used in research; SA‐β‐gal, senescence‐associated beta‐galactosidase; SKBR‐3 cells, human breast cancer cell line; Wistar rats, strain of rats used in research. Symbols: ↑ increase, ↓ decrease.

In vivo studies have also shown promising results regarding the anticancer efficacy of diosmin (Table 2). It has been reported that diosmin was investigated as a chemopreventive agent in a rat model of breast cancer (Şöhretoğlu et al., 2021; Tanaka, Makita, Ohnishi, et al., 1997). It has also been found that diosmin treatment significantly reduced the incidence of breast tumors and decreased the levels of various tumor markers, including carcinoembryonic antigen (CEA) and lactate dehydrogenase (LDH). In addition, diosmin treatment was associated with a significant increase in the levels of antioxidant enzymes, such as superoxide dismutase (SOD) and catalase, indicating that diosmin may exert its anticancer effects through its antioxidant properties (Tanrikulu et al., 2013). The combination treatment of diosmin was also associated with a significant decrease in the levels of various inflammatory cytokines, such as interleukin‐6 (IL‐6) and tumor necrosis factor‐alpha (TNF‐α), indicating that diosmin may exert its anticancer effects by modulating the immune system (Al‐Khayri et al., 2022). Diosmin was investigated as a potential therapy for hepatocellular carcinoma (HCC) in a rat model. The study found that diosmin treatment significantly reduced the growth and size of HCC tumors and decreased the levels of various tumor markers, including alpha‐fetoprotein (AFP) and vascular endothelial growth factor (VEGF). In addition, diosmin treatment was associated with a significant increase in the levels of apoptotic markers, such as caspase‐3 and Bax, indicating that diosmin may exert its anticancer effects by inducing apoptosis (Perumal et al., 2018). Overall, the results of these studies suggest that diosmin has promising anticancer potential and may be a useful adjuvant therapy for the treatment of various cancers. However, further studies are needed to elucidate the underlying mechanisms of the anticancer effects of diosmin and to determine the optimal dosing and administration regimens for diosmin in cancer therapy.

### 4.5 Synergistic anticancer effects of diosmin in combination with conventional chemotherapy and other bioactive natural compounds

The combination of natural compounds like diosmin with conventional chemotherapy has emerged as a promising strategy in cancer treatment; this approach leverages the unique mechanisms of each agent, potentially enhancing efficacy while mitigating side effects (Castañeda et al., 2022). The combination of diosmin has led to the investigation of diosmin as a potential adjuvant therapy in cancer treatment; this suggests that diosmin may have a synergistic effect when used in combination with conventional cancer treatments, potentially enhancing their efficacy and reducing the risk of drug resistance. Adjuvant therapy aims to eliminate any residual cancer cells and reduce the risk of cancer recurrence (Naso et al., 2016; Şöhretoğlu et al., 2021). Diosmin showed its anti‐carcinogenic activity in many types of cancer, where it displays anti‐tumor activity through multiple signaling pathways and inhibition, including PI3K‐Akt, NF‐κB, and VEGF (Helmy et al., 2020); in the case of colorectal cancer, the combination of diosmin and dactolisib suppresses cancer cell proliferation and induces apoptosis by downregulating PI3K/AKT/mTOR/NF‐κB signaling (Helmy et al., 2020). In addition to chemotherapy drugs, diosmin has also been studied in combination with other natural compounds for potential synergistic effects. The effect of diosmin in combination with curcumin, a compound found in turmeric with known anti‐cancer properties, has been investigated in an in vitro study against breast cancer and oral cancer. These studies found that the combination of diosmin and curcumin had a synergistic effect in inhibiting cancer cell growth and inducing apoptosis, with a greater effect than either compound alone (Li et al., 2022; Musyayyadah et al., 2021). Similarly, another study investigated the effect of diosmin in combination with quercetin, a flavonoid with known anti‐cancer properties. The study found that the combination of diosmin and quercetin had a synergistic effect in inhibiting cancer cell growth and inducing apoptosis, with a greater effect than either compound alone (Schlachterman et al., 2008). In a recent study, the synergistic anticancer effects of diosmin and PGV‐1 (Pentagamavunone‐1), a curcumin analog co‐treatment, on 4 T1 cancer cells were evaluated. This combination was found to target key cell cycle regulatory proteins, namely CDK1, KIF11, and AURKA, in triple‐negative breast cancer (TNBC). The co‐treatment effectively induced mitotic catastrophe, a type of cell death, and enhanced senescence, a form of permanent cell cycle arrest. These mechanisms collectively contribute to the increased cytotoxicity observed in the cancer cells, indicating the potential of this combination as an effective therapeutic approach for TNBC (Musyayyadah et al., 2021). Berberine and diosmin combination forms nano‐micelles, which showed anticancer activity in hepatocellular carcinoma mice by downregulating angiogenesis and TNF‐α, COX‐2, NF‐κB, and TNF‐α, and through the initiation of apoptosis (Zheng et al., 2020). The diosmetin in non‐small cell lung cancer enhances the effect and efficacy of the cytostatic drug paclitaxel, while the production of reactive oxygen species and disruption of the PI3K/Akt/GSK‐3β pathway cause apoptosis in the NSCLC cells and decrease the stability of the Nrf2 gene (Chen, Wu, et al., 2019). In another recent study investigating colon cancer, the combined effect of Diosmin and Naringenin (NR), known as DiNar, was examined on colon cancer cell lines HCT116 and SW480 (Zeya et al., 2022). This research focused on assessing DiNar's impact on cell proliferation, apoptosis, and inflammatory pathways, and key findings included DiNar's ability to synergistically induce cytotoxicity, leading to increased apoptosis and cell cycle arrest in the G0/G1 phase. Additionally, DiNar effectively regulated the expression of apoptotic and inflammatory markers. The study suggests DiNar could be a potential novel chemotherapeutic approach for colon cancer (Zeya et al., 2022). The combination of diosmin with conventional chemotherapy and other bioactive natural compounds presents a synergistic approach that enhances the therapeutic efficacy against various cancers. By targeting multiple pathways, this combination strategy not only improves the effectiveness of treatment but also offers a potential reduction in side effects, thereby improving patient outcomes in cancer therapy. Further clinical trials and in‐depth mechanistic studies are essential to fully harnessing the benefits of this combinatory approach in oncology.

Table 3 presents a comprehensive overview of recent studies focusing on the synergistic anticancer effects of diosmin when combined with conventional chemotherapy agents and other bioactive natural compounds.

**TABLE 3:** Synergistic anticancer effects of Diosmin in combination with chemotherapy and bioactive natural compounds.

Combined treatment	Cancer type	Synergistic effects	Mechanisms of action	References
Diosmin + Dactolisib	Colorectal Cancer	↓ Cancer cell proliferation ↑ Apoptosis	↓ PI3K/AKT/mTOR/NF‐κB ↑ Apoptosis	Helmy et al. (2020)
Diosmin + Paclitaxel	Non‐Small Cell Lung Cancer	↑ Drug efficacy ↑ Apoptosis	↓ PI3K/Akt/GSK‐3β ↓ Nrf2 gene stability; ↑ apoptosis	Chen, Wu, et al. (2019)
Diosmin + Curcumin	Breast Cancer Oral Cancer	↑ Inhibition of cancer ↑ Cell growth ↑ Apoptosis	Synergistic enhancement of apoptosis More effective than either compound alone	Li et al. (2022); Musyayyadah et al. (2021)
Diosmin + Quercetin	Breast cancer	Inhibition of cancer cell growth ↑ Apoptosis	Greater effect than either compound alone Synergistic apoptosis induction	Schlachterman et al. (2008).
Diosmin + PGV‐1 (Pentagamavunone‐1)	Triple‐Negative Breast Cancer	Mitotic catastrophe ↑ Senescence ↑ Cytotoxicity	Targeting CDK1, KIF11, and AURKA; ↑ Cell cycle arrest; ↑ cell death	Musyayyadah et al. (2021).
Diosmin + Berberine	Hepatocellular Carcinoma	↑ Anticancer activity ↑ Apoptosis induction	↓ Angiogenesis, ↓ TNF‐α, ↓ COX‐2, ↓ NF‐κB; ↑ apoptosis	Zheng et al. (2020)
Diosmin + Naringenin (DiNar)	Colon Cancer	↑ Cytotoxicity ↑ Apoptosis ↑ Cell cycle arrest	Regulating expression of apoptotic and inflammatory markers; ↑cancer cell death	Zeya et al. (2022)

Abbreviations: COX‐2, cyclooxygenase‐2; G0/G1, Gap 0/Gap 1 phase in cell cycle; GSK‐3β, glycogen synthase kinase 3 beta; mTOR, Mammalian target of rapamycin; NF‐κB, nuclear factor kappa B; NSCLC, non‐Small Cell Lung Cancer; PI3K, Phosphoinositide 3‐Kinase; ROS, Reactive Oxygen Species; TNF‐α, tumor necrosis factor alpha. Symbols: ↑ increase, ↓ decrease.

## 5 OTHER THERAPEUTIC APPLICATIONS OF DIOSMIN

### 5.1 Venotonic effect

Venotonics are active substances used in the treatment of venous disorders, having the effect of increasing the tonicity of the venous walls, and many bioactive compounds have an adjuvant venotonic effect (Gwozdzinski et al., 2024). The mechanisms of venotonic action of diosmin include increased lymphatic drainage, inhibition of inflammatory reactions, reduced capillary permeability, improvement of venous tone, and protection of capillary bed microcirculation. Some flavonoids, including diosmin, are promising inhibitors of thromboxane A2 (TxA2)7, and prostaglandin E2 (PGE2) also showed inhibition of leukocyte migration, activation, and adhesion. Diosmin also reduces neutrophil activation and causes a promising decrease in the level of plasma endothelial adhesion molecules, which protect against microcirculatory damage (Jantet, 2002).

### 5.2 Antioxidant

Various diseases are linked with oxidative stress, including diabetes, neuronal cell injury, cerebral ischemia–reperfusion injury, cancer, and hypoxia. Diosmin protects against cerebral ischemia/reperfusion injury by activating the JAK2/STAT3 signal pathway in mice (Liu et al., 2014). The study observed increased expression of phosphorylated JAK2 and STAT3, along with decreased neurological deficit, brain water content, and infarct volume in mice treated with high doses of Diosmin compared to controls. This suggests Diosmin's potential as a therapeutic agent for cerebral I/R injury by modulating apoptotic pathways via JAK2/STAT3 activation. (Liu et al., 2014) Diosmin has been found to exhibit antioxidant activity in many rat models and to stimulate human neutrophils. Diosmin offers a lot of therapeutic effects for diseases characterized by oxidative stress (Chikara et al., 2018; Khosravi et al., 2019; Poznyak et al., 2020). The diabetic condition is caused by a decrease in antioxidants in the body (Quispe et al., 2022). According to previously reported studies in diabetic model rats, a low level of antioxidants such as reduced glutathione (GSH), vitamin C, and vitamin E was found with low antioxidant enzyme activities, such as superoxide dismutase (SOD), glutathione‐S‐transferase (GST), glutathione peroxidase (GPx) catalase (CAT), and plasma insulin, while a high lipid peroxidation hyperglycemia was also seen (Zhang et al., 2020). In such cases, diosmin was found to lower the lipid peroxidation and enhance the glycemic and antioxidant status of the body (Srinivasan & Pari, 2012; Zhang et al., 2020). In another study, the diosmin anti‐hypertensive drug activity was reported in deoxycorticosterone acetate (DOCA)‐salt‐induced hypertensive rats models, where the low level of enzymatic condition and the high level of lipid peroxidation products were found in the tissue and blood plasma of the kidney, aorta, heart and liver. With the treatment of diosmin, the enzymatic condition was restored while the lipid peroxidation was reduced to its normal value (Adouani et al., 2020; Bogucka‐Kocka et al., 2013; Bozdağ & Eraslan, 2020). An excessive amount of iron in the body causes oxidative stress, lipid peroxidation, reactive oxygen species (ROS), and tissue necrosis, which indicate hepatocyte membrane damage (Perumal et al., 2018). Diosmin, a good hepatoprotective drug, considerably normalized this condition because it decreases oxidative stress, adds to the correction of dyslipidemia, and maintains membrane integrity.

### 5.3 Antibacterial

Many bacteria and fungi have developed resistance to the majority of available antibiotics; therefore, new potential compounds are needed to have antibacterial activity. In this case, medicinal plants are the hope for discovering new effective antibacterial agents; such bioactive compounds are isolated from plants that have effective activity against yeast, fungi, and bacteria (Keita et al., 2022). Diosmin can damage microbial membrane structure, inhibit the synthesis of peptidoglycans, interfere with insects called the phytochemicals quorum sensing (QS) and change the bacterial membrane hydrophobicity surfaces; it showed antibacterial activity against many bacteria like *Escherichia coli*, *Staphylococcus aureus* and *Pseudomonas putida* due to changes in the permeability of cell membrane, suppression of the function of the respiratory enzyme function, generation of free radicals, causing the cavities on the bacterial cell wall, various thiol‐containing enzymes inactivation, obstruction of transduction and changes in the cell membrane of the bacterial cell (Kilit et al., 2021; Sahu et al., 2016). The flavonoids' antibacterial properties are linked to the inhibition of ATP synthase. In the case of antibacterial activity against *E. coli*, the compound diosmin partially inhibits ATP synthesis, which causes the death of *Escherichia coli* (Chinnam et al., 2010). Diosmin has been virtually docked with the *Mycobacterium tuberculosis* enzymes LdtMt2 and LdtMt1, which are involved in their cell wall biosynthesis (Pushkaran et al., 2019). Date palm methanolic extracts showed broad‐spectrum antibacterial activity against various pathogenic bacteria like *Staphylococcus aureus*, *E. coli*, *Bacillus subtilis*, *Streptococcus pneumoniae*, and *Klebsiella pneumonia*, which contain the compound diosmin (Alshwyeh, 2020). Diosmin isolated from *Phlomis viscosa* helped in wound healing by preventing the growth of pathogenic bacteria *Pseudomonas aeruginosa* and *Staphylococcus aureus* and downregulate the inflammatory cytokines IL‐6/8 expression (Yarmolinsky et al., 2019).

### 5.4 Hepatoprotective

Angiogenesis, intrapulmonary vasodilation, hypoxia, and inflammation characterize a serious condition of hepatic cirrhosis and hepatopulmonary syndrome (HPS). Diosmin treatment also restored HPS development in an induced rat model of chronic bile duct ligation (CBDL) by changing TNF/VEGF, FGF‐1/ANG‐2, and IGF‐1/PI3K/AKT signaling pathways. Diosmin, through modification of the p38‐MAPK/NF‐B/iNOS and Keap‐1/NRF2 signaling pathways, decreased BDL‐induced liver abnormalities. Similarly, the pentoxifylline and diosmin combination caused a change in the NF‐kB/p65/p38‐MAPK and Keap‐1/Nrf‐2/GSH signaling pathways and reduced BDL‐induced liver cirrhosis. Diosmin also increases the production of the antioxidant, anti‐fibrotic, and anti‐inflammatory compound cytoglobin and accelerate the function of the eNOS gene with suppression of the ROS‐induced p38 MAPK and promotion of NRF2/Keap‐1 pathways. Diosmin upregulates the production of HO‐1 and SOD target genes by binding to the ARE sequence and activating NRF2 to increase transcriptional activity by entering the nucleus of the cell. Diosmin also activates the signaling pathways of NF‐kB, p52, and iNOS by triggering p38 MAPK (Imam et al., 2015; Pari & Srinivasan, 2010).

### 5.5 Anti‐inflammatory

Inflammation causes many diseases, including allergies, asthma, diabetes, cancer, arthritis, autoimmune diseases, and atherosclerosis. Different inflammatory biomarker levels are used for the detection of inflammation, and these include eosinophils, basophils, platelets, macrophages, and neutrophil immune cells. Some soluble mediators such as cytokines (TNF‐α, TGF‐ß, IFN‐γ, IL‐1, IL‐2, and IL‐6), chemokines, adhesion molecules, and surface receptors such as P‐selectin, E‐selectin, and L‐selectins, NF‐kB (coagulation factor fibrinogen, C‐reactive protein, and complement factors), and cytokines (TGF‐ß, IFN‐γ, and TNF‐α) (Ali et al., 2021; Yao et al., 2021). According to another study in the case of attenuated inflammatory responses, diosmin suppresses the activity of MAPKs and NF‐κB singling pathways in lipopolysaccharide (LPS)‐triggered macrophages (RAW264.7) (Berkoz, 2019). The lung injury is caused by LPS (lipopolysaccharide) expression. The compound has been reported during LPS‐induced inflammation to reduce the expression of IL‐17+, IL‐2+, CD8+, and CD4+ receptors by stimulating LPS and downregulating the NF‐κB signaling pathway (Imam et al., 2015). The compound has also been shown to inhibit IKKβ and block NF‐κB activation. Diosmin also interacts with the hydrophobic residues Val29, Leu21, I1e165, and Val152 of IKKβ and inhibits its activity. Diosmin also ameliorates cerulein‐induced acute pancreatitis by decreasing the level of IL‐6, TNF, IL‐1 pro‐inflammatory cytokines, trypsinogen activation peptide, iNOS, and myeloperoxidase activity and NF‐κB signaling pathway downregulation. (Yu et al., 2014). During the meningitis disease with *Streptococcus pneumoniae*, diosmin reduced the condition of neuronal apoptosis and neuro‐inflammation by regulating the phosphoinositide‐3‐kinase (PI3K)/AKT/Nuclear factor‐кB (NF‐кB) signaling pathway (Zhang et al., 2019). Another study showed that plant *Phlomis viscose* base‐derived diosmin reduces the secretions of many inflammatory‐causing cytokines (Yarmolinsky et al., 2019). Diosmin caused the inactivation of the NLRP3 inflammasome in lung tissues by promoting the Nrf2 expression accompanied by target gene HO‐1 and by controlling the increased levels of ROS, MPO, and MDA (Xing‐Yan et al., 2018). In the case of acetic acid‐induced ulcerative colitis, the compound diosmin changes the levels of cyclooxygenase‐2 (COX‐II) and tumor necrosis factor (TNF‐α) in a dose‐dependent manner (Shalkami et al., 2018).

### 5.6 Anti‐diabetes

Diabetes is a disease (chronic) caused by abnormal lipid metabolism, protein and glucose levels, and a decrease or lack of insulin activity (“Global, regional, and national burden of diabetes from 1990 to 2021, with projections of prevalence to 2050: a systematic analysis for the Global Burden of Disease Study 2021,” 2023). Diosmin possesses anti‐hyperglycemic activity and improves glycemic control. Diosmin has a dose‐dependent effect on the plasma glucose level; the oral administration of diosmin showed a decrease in glycosylated hemoglobin while raising plasma insulin and hemoglobin (Perumal et al., 2018). Diosmin reduced diabetes by targeting the PTP‐1B signaling pathway, and the α‐glucosidase enzyme also suppressed two liver enzymes, glucose‐6‐phosphate dehydrogenase and hexokinase (Chen, Wang, et al., 2019). Diosmin has effects on the lipid metabolism of patients with type 2 diabetes by inhibiting hepatic cholesterol synthesis and reducing plasma lipoproteins (Farmer, 2008; Huwait & Mobashir, 2022). Recent studies showed that plasma lipid, plasma lipoprotein, and tissue lipid (TGs, PLs, cholesterol, and FFAs) can be normalized with the treatment of diosmin. Diosmin acting on the adrenal gland also promotes the secretion of β‐endorphin and stimulates opioid receptor expression, which helps to control the condition of hyperglycemia in STZ‐induced rats models (Hsu et al., 2017). According to another reported study, in STZ‐induced diabetic rats, the compound diosmin was found to reduce the levels of fructose‐1,6‐bisphosphatase, glycosylated hemoglobin, and glucose‐6‐phosphatase (G6P) while increasing the levels of plasma insulin, hemoglobin, glucose‐6‐phosphate dehydrogenase (G6PD), and hexokinase (Pari & Srinivasan, 2010). Diosmin was also found to improve the resistance to insulin, the production of lipoprotein and uric acid, a decrease in blood pressure, the albumin/creatinine ratio, and the albumin excretion rate (El‐Fawal et al., 2018). Diosmin also improves lactate dehydrogenase, ameliorates cardiac creatine kinase enzyme expression, and improves insulin resistance in diabetic patients. In alloxan‐induced diabetic Wistar mice, the treatment of diosmin considerably normalized NF‐kB, which is an important factor for many inflammatory illnesses and the pathogenesis of diabetic neuropathy (Ahmed et al., 2016).

### 5.7 Cardioprotective

**FIGURE 4:**
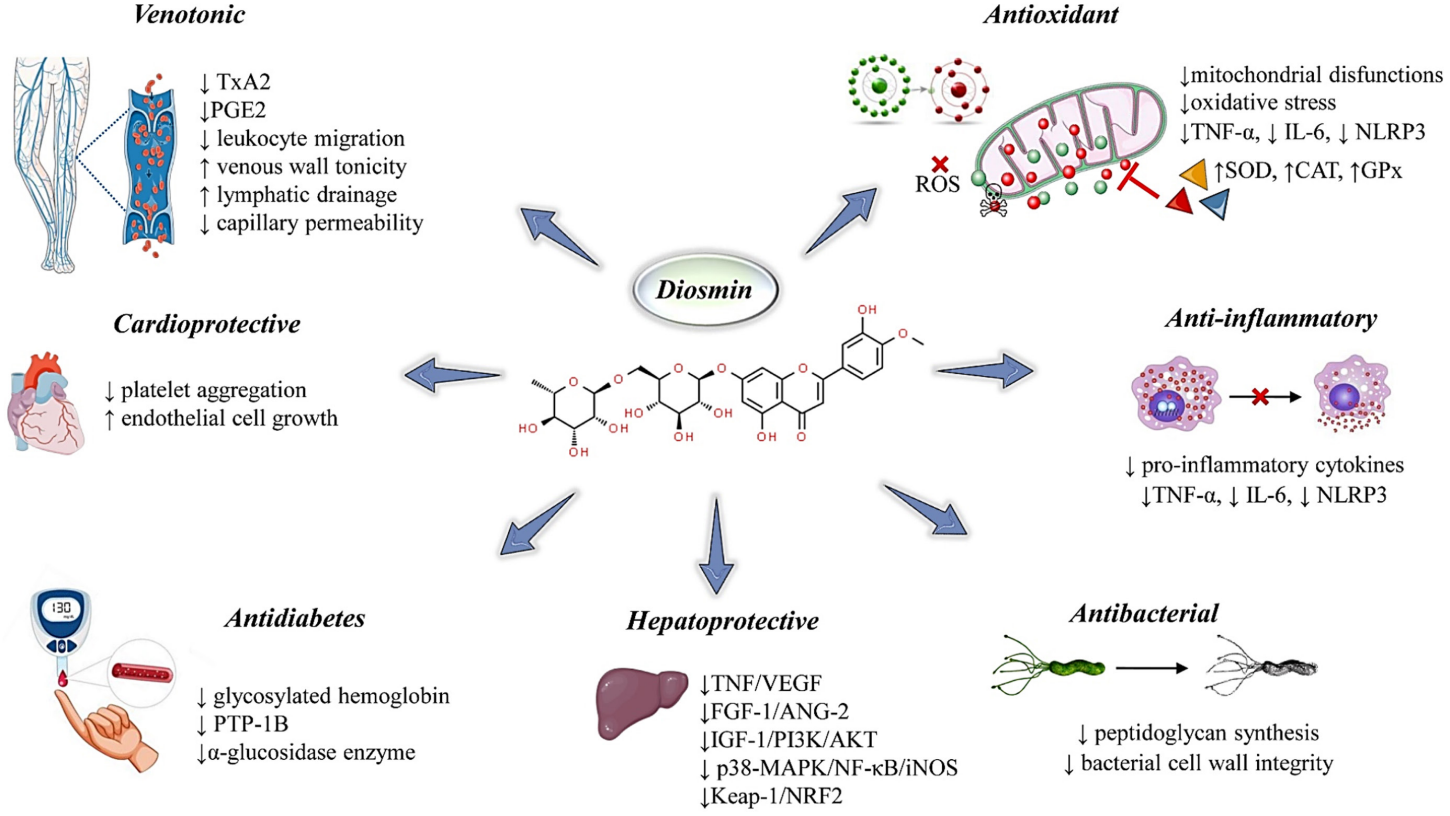
Pharmacological effects of diosmin: a biochemical and cellular overview. The figure illustrates the diverse pharmacological properties of diosmin, a naturally occurring flavonoid glycoside. In the context of venous health, diosmin exhibits venotonic effects by reducing thromboxane A2 (TxA2) and prostaglandin E2 (PGE2) levels, decreasing leukocyte migration, enhancing venous wall tonicity and lymphatic drainage, and reducing capillary permeability. Cardiovascular benefits are highlighted by its ability to diminish platelet aggregation and promote endothelial cell growth. Diosmin's antidiabetic potential is demonstrated through the reduction of glycosylated hemoglobin, protein tyrosine phosphatase 1B (PTP‐1B), and alpha‐glucosidase enzyme activity. Hepatoprotective actions are conveyed by downregulating tumor necrosis factor (TNF)/vascular endothelial growth factor (VEGF), fibroblast growth factor‐1 (FGF‐1)/angiopoietin‐2 (ANG‐2), insulin‐like growth factor‐1 (IGF‐1)/phosphoinositide 3‐kinase (PI3K)/AKT signaling, and inhibiting p38 mitogen‐activated protein kinases (MAPK)/nuclear factor kappa‐light‐chain‐enhancer of activated B cells (NF‐κB)/inducible nitric oxide synthase (iNOS) and Kelch‐like ECH‐associated protein 1 (Keap‐1)/nuclear factor (erythroid‐derived 2)‐like 2 (NRF2) pathways. Antioxidant properties are evidenced by the reduction of mitochondrial dysfunctions and oxidative stress, along with the increase in superoxide dismutase (SOD), catalase (CAT), and glutathione peroxidase (GPx) activities. Diosmin's anti‐inflammatory capabilities are supported by its suppression of pro‐inflammatory cytokines, including TNF‐α, interleukin‐6 (IL‐6), and NOD‐like receptor protein 3 (NLRP3). Lastly, its antibacterial effect is attributed to the impairment of peptidoglycan synthesis and the compromise of bacterial cell wall integrity. Symbols: ↑ increase, ↓ decrease.

The diet contains more flavonoid contents to help in the restoration of cardiovascular diseases, and diosmin was found to have an anti‐platelet activity where its sulfation enhances the overall binding to the heparin‐binding site involving the N‐terminal residues helix D and A, leading to the development of a new significant candidate for anti‐thrombin (Rashid et al., 2014). The activity of diosmin was also observed in the rat's model with venous thrombosis with the change in protein profile. The compound diosmin targets the centrosome‐associated protein 350 (CEP350) for the improvement of endothelial cell growth (Cheng et al., 2017). In nitric oxide synthesis inhibitor L‐NAME‐induced rat models, the antihypertensive effect of diosmin is studied by protecting the rats from hyaline arteriopathy, fibrinoid necrosis, and myocardial infarctions caused by L‐NAME. In the case of anti‐hypertension, the main function of diosmin was demonstrated as the elimination of superoxide anions (Paredes et al., 2018). In isoproterenol‐induced myocardial‐infarcted rats' models, diosmin has been found to reduce and restrict lipid metabolism abnormalities, decrease plasma lipid peroxidation and serum cardiac marker enzyme production, resulting in anti‐hyperlipidemia, and help in cardioprotection. Furthermore, isoproterenol conditions increase calcium ion, mitochondrial lipid peroxidation, and cardiac diagnostic markers and decrease the expression of antioxidant enzymes, which are effectively treated with diosmin (Fattori et al., 2020). Isoproterenol‐induced myocardial infarction pathogenesis in rats was caused by ATPase dysfunction, electrolyte imbalance, and left ventricular hypertrophy (LVH). Diosmin treatment was found to effectively compromise these pathological changes (Stansfield et al., 2014). Diosmin controls the elevation of lipid peroxidation and prevents the dysfunction of antioxidant enzyme expression. The cardiovascular complications in rats have been improved with the treatment of diosmin, as demonstrated by the amelioration of systolic and diastolic blood pressure (BP) and electrocardiogram (ECG) parameters by inhibiting inflammation and oxidative stress (El‐Fawal et al., 2018). Diosmin has been reported to give protection against hyaline arteriopathy, fibrinoid necrosis, and myocardial infarctions caused by reducing plasma lipid peroxidation, lipid metabolite alterations, and serum cardiac marker enzyme production. In the urine, diosmin helps in lowering the value of pH, protein, kidney weight, calcium, and phosphorus, while in blood, it helps in lowering the quantity of magnesium, creatinine, uric acid, and sodium. The most important data regarding other biomedical applications are summarized in Figure 4 and Table 4.

**TABLE 4:** Overview of diosmin's therapeutic applications and mechanisms.

Pharmacological properties	Effects and outcomes	Mechanisms	References
Venotonic	↑ Venous wall tonicity ↑ Lymphatic drainage ↓ Capillary permeability ↑ Microcirculation protection	↓ TxA2 ↓ PGE2 ↓ Leukocyte migration/activation/adhesion ↓ Plasma endothelial adhesion molecules	Jantet (2002)
Antioxidant	↓ Oxidative stress in diseases (diabetes, cerebral ischemia, cancer) ↑ Neutrophil stimulation	↑ Antioxidant enzymes (SOD, GST, GPx, CAT) ↓ ROS, ↓ lipid peroxidation ↑ JAK2/STAT3 ↑ Membrane integrity maintenance	Liu et al. (2014); Srinivasan and Pari (2012); Zhang et al. (2020)
Antibacterial	Effective against pathogens (*E. coli, S. aureus, P. putida*) ↑ Microbial membrane damage	↓ Peptidoglycan synthesis, interference with QS changes in membrane hydrophobicity ↓ Bacterial cell wall integrity	Alshwyeh (2020); Kilit et al. (2021); Sahu et al. (2016); Yarmolinsky et al. (2019)
Hepatoprotective	Restores liver function in hepatic cirrhosis and HPS.	Modulation of signaling pathways ↓ TNF/VEGF ↓ FGF‐1/ANG‐2 ↓ IGF‐1/PI3K/AKT ↓ p38‐MAPK/NF‐κB/iNOS ↓ Keap‐1/NRF2	Imam et al. (2015); Pari and Srinivasan (2010)
Anti‐inflammatory	↓ Inflammation in various diseases (allergies, asthma, diabetes)	↓ MAPKs, ↓ NF‐κB ↓ Pro‐inflammatory cytokines ↓ TNF‐α, ↓ IL‐6 ↓ NLRP3 inflammasome	Berkoz (2019); Shalkami et al. (2018); Xing‐Yan et al. (2018); Yu et al. (2014)
Anti‐diabetes	↓ Hyperglycemia ↑ Glycemic control	↑ Plasma insulin ↓ Glycosylated hemoglobin ↓ PTP‐1B ↓ α‐Glucosidase enzyme	Farmer (2008); Hsu et al. (2017); Huwait and Mobashir (2022)
Cardioprotective	↓ Risk of myocardial infarction ↓ Lipid peroxidation	↓ Platelet aggregation ↑ Endothelial cell growth Modulation of lipid metabolism	Cheng et al. (2017); El‐Fawal et al. (2018); Fattori et al. (2020)

Abbreviations: ANG‐2, angiopoietin‐2; CAT, catalase; CBDL, chronic bile duct ligation; CEP350, centrosome‐associated protein 350; COX‐II, cyclooxygenase‐2; DOCA, deoxycorticosterone acetate; ECG, electrocardiogram; FGF‐1, fibroblast growth factor 1; GPx, glutathione peroxidase; GSH, reduced glutathione; GST, glutathione‐S‐transferase; HPS, hepatopulmonary syndrome; IGF‐1, insulin‐like growth factor 1; IL‐1, interleukin 1; IL‐2, interleukin 2; IL‐6, interleukin 6; IL‐8, interleukin 8; IL‐17, interleukin 17; IKKβ, IκB kinase β; IκBα, inhibitor of κBα; iNOS, inducible nitric oxide synthase; LPS, lipopolysaccharide; LVH, left ventricular hypertrophy; MAPK, mitogen‐activated protein kinase; MDA, malondialdehyde; MPO, myeloperoxidase; NF‐κB, nuclear factor kappa‐light‐chain‐enhancer of activated B cells; NRF2, nuclear factor erythroid 2–related factor 2; NLRP3, NOD‐, LRR‐ and pyrin domain‐containing protein 3; PI3K, phosphoinositide 3‐kinase; PGE2, prostaglandin E2; PI3K/AKT, phosphoinositide 3‐kinase/protein kinase B; PTP‐1B, protein tyrosine phosphatase 1B; QS, quorum sensing; RelA, v‐rel avian reticuloendotheliosis viral oncogene homolog A; ROS, reactive oxygen species; SOD, superoxide dismutase; TGF‐ß, transforming growth factor beta; TNF, tumor necrosis factor; TxA2, thromboxane A2; VEGF, vascular endothelial growth factor.

## 6 TOXICITY AND SAFETY DATA OF DIOSMIN

Diosmin has been shown to be safe and well‐tolerated in numerous preclinical and clinical studies. In preclinical toxicology studies, diosmin did not show any significant toxic effects, even at high doses. In clinical studies, diosmin has been well tolerated with few reported adverse effects. The most commonly reported adverse effects associated with diosmin use are gastrointestinal disturbances such as nausea, vomiting, and diarrhea, which are usually mild and transient. Headache, dizziness, and skin rashes have also been reported in some cases. These adverse effects are usually self‐limiting and resolve spontaneously (Cazaubon et al., 2021; Gupta & Raina, 1998). Diosmin has not been associated with any serious adverse effects or toxicity in clinical trials. In studies of the safety data from clinical practices involving more cancer patients and other disease patients, no serious adverse effects or drug interactions were reported. In other studies, diosmin was found to have no significant effect on liver or kidney function, even when used at high doses (Eraslan et al., 2017; Zou et al., 2022). In terms of drug interactions, diosmin has not been shown to interact with other drugs or to affect the pharmacokinetics of co‐administered drugs.

In preclinical toxicology assessments, diosmin was administered to Sprague–Dawley rats at doses of 75, 150, and 300 mg/kg/day over 90 days. The no‐observed‐adverse‐effect level (NOAEL) was determined to be 300 mg/kg/day, indicating minimal toxicity at this dosage (Xu et al., 2012). In diabetic rat models, diosmin dosages up to 100 mg/kg/day effectively reduced oxidative stress and improved biochemical markers without significant adverse effects (Pari & Srinivasan, 2010).

Clinical studies have confirmed the safety of diosmin at doses up to 2000 mg/day. A randomized, placebo‐controlled trial indicated no significant adverse effects at doses of 1000 mg and 2000 mg per day over four months, with normal blood parameters and no clinically relevant deviations from baseline values (Staniewska, 2016). Additionally, in patients with chronic venous disease, a dose of 450 mg once daily for eight weeks significantly improved symptoms without any treatment‐related adverse events (Serra et al., 2021).

In conclusion, diosmin appears to be safe and well‐tolerated, with few reported adverse effects. It has not been associated with any serious adverse effects or toxicity in clinical trials. However, as with any drug, caution should be exercised when using diosmin, especially in patients who are taking other medications or who have underlying medical conditions.

## 7 DIOSMIN IN MEDICINE: CLINICAL PERSPECTIVES, LIMITATIONS, AND FUTURE DIRECTIONS

### 7.1 Clinical evidence

Recent clinical studies have explored the anticancer potential of diosmin, particularly in its combination with chemotherapy. A notable study in 2015 investigated diosmin's effects in patients with advanced non‐small cell lung cancer. This study, conducted by Qi et al. (2015) revealed that diosmin, when combined with chemotherapy, significantly enhanced progression‐free and overall survival rates compared to chemotherapy alone. Similarly, research by Shafique et al. (2019) on gastric cancer patients demonstrated that diosmin, in conjunction with chemotherapy, significantly improved overall survival rates over chemotherapy alone (Shafique et al., 2019).These findings suggest a potential synergistic effect of diosmin when used in combination with conventional cancer treatments. Beyond cancer, diosmin has been investigated for its efficacy in treating other medical conditions. Serra et al. (2021) conducted a randomized, double‐blind, placebo‐controlled trial assessing diosmin's impact on chronic venous disease symptoms in women. The results indicated significant improvements in symptoms such as leg pain, edema, and cramps with diosmin treatment compared to a placebo. Additionally, a study by Zheng et al. (2020) explored the effects of diosmin on cognitive function in the elderly, finding notable improvements in cognitive performance relative to placebo recipients.

### 7.2 Limitations and clinical gaps

Despite promising preclinical data, there is a notable scarcity of clinical trials specifically focusing on diosmin's anticancer properties; the current clinical evidence primarily stems from in vitro or animal studies, which might not fully represent the compound's effects in human populations. Also, existing studies on diosmin show significant variability in terms of dosages, administration methods, and study populations, and this diversity in study design complicates the process of drawing consistent and generalizable conclusions about diosmin's therapeutic potential. The majority of research on diosmin's anticancer effects is concentrated on a limited range of cancer types, limiting our understanding of its potential efficacy across a broader spectrum of oncological conditions. There is a deficiency in long‐term studies examining the extended effects and potential long‐term advantages or risks associated with diosmin use, which are important for a comprehensive understanding of its safety profile. The existing literature lacks studies that directly compare diosmin with other anticancer agents; comparative studies are essential for situating diosmin within the current therapeutic landscape and for evaluating its relative efficacy and safety. Studies often utilize diosmin from varying sources, and the purity levels of diosmin can differ, potentially influencing study outcomes and complicating comparisons across different research efforts.

### 7.3 Future directions

Longitudinal studies are essential to assess the long‐term safety and efficacy of Diosmin, especially when used as a chronic treatment in cancer and other conditions. Understanding the long‐term implications of Diosmin use is important for its integration into standard treatment protocols. In‐depth mechanistic studies are required to unravel the molecular pathways through which Diosmin exerts its anticancer and therapeutic effects; this would involve exploring its interactions at the cellular and molecular levels, potentially using advanced genomic, proteomic, and metabolomic techniques. Further research into the pharmacokinetics and pharmacodynamics of diosmin would provide valuable information on its absorption, distribution, metabolism, and excretion, which are critical for optimizing dosing regimens and minimizing potential drug interactions. Incorporating patient‐reported outcomes in future studies would provide a more holistic view of Diosmin's impact on patients' quality of life, particularly in the context of chronic diseases. Investigating different formulations and delivery methods of diosmin could enhance its bioavailability and therapeutic efficacy; this may include the development of novel drug delivery systems or combination therapies. Also, more cost‐effectiveness analyses of Diosmin in various therapeutic applications would be valuable, especially considering the economic burden of chronic diseases like cancer.

## 8 CONCLUSIONS

Diosmin, a flavonoid derived from citrus fruits, has garnered significant attention for its potential anticancer properties. This review consolidates evidence demonstrating diosmin's ability to interfere with various cancer pathways, including the induction of apoptosis, inhibition of angiogenesis, suppression of metastasis, and chemopreventive activity. Its mechanisms of action involve the modulation of critical pathways such as PI3K‐AKT/MDM2, NF‐κB, and VEGF.

Diosmin's synergistic effects when used in combination with conventional chemotherapeutic agents and other bioactive compounds suggest a promising enhancement in cancer treatment efficacy. Moreover, diosmin's therapeutic benefits extend beyond oncology, encompassing treatment for venous disorders due to its venotonic and vascular protective properties. It also shows potential for managing oxidative stress, inflammatory conditions, microbial infections, and metabolic and cardiovascular disorders. Clinical studies have indicated diosmin's favorable safety and tolerability profile, characterized by minimal side effects, predominantly gastrointestinal, even at higher doses. This aspect is fundamental for its potential integration into long‐term treatment regimens for chronic conditions. However, despite the promising data, there is a necessity for extensive clinical trials to further validate its long‐term impacts and broaden our understanding of its therapeutic potential. Future research should focus on elucidating the detailed molecular mechanisms underlying diosmin's actions, refining dosing strategies, and exploring advanced delivery systems to optimize its therapeutic efficacy. In conclusion, diosmin presents substantial promise as a therapeutic agent, particularly in cancer treatment, and its potential to enhance the efficacy of existing treatments offers a novel perspective in oncological management. The extensive application across various medical conditions reinforces its value as a versatile bioactive molecule in future medical treatment strategies.

## AUTHOR CONTRIBUTIONS

**Lubna Rahman:** Data curation (equal); investigation (equal); methodology (equal); writing – original draft (equal); writing – review and editing (equal). **Ali Talha Khalil:** Data curation (equal); investigation (equal); methodology (equal); writing – original draft (equal); writing – review and editing (equal). **Syed Ahsan Shahid:** Data curation (equal); investigation (equal); methodology (equal); writing – original draft (equal); writing – review and editing (equal). **Zabta Khan Shinwari:** Data curation (equal); investigation (equal); methodology (equal); supervision (equal); validation (equal); visualization (equal); writing – original draft (equal); writing – review and editing (equal). **Zainab M. Almarhoon:** Data curation (equal); investigation (equal); methodology (equal); writing – original draft (equal); writing – review and editing (equal). **Amnah Alalmaie:** Data curation (equal); investigation (equal); methodology (equal); writing – original draft (equal); writing – review and editing (equal). **Javad Sharifi‐Rad:** Conceptualization (equal); data curation (equal); formal analysis (equal); investigation (equal); methodology (equal); project administration (equal); supervision (equal); validation (equal); visualization (equal); writing – original draft (equal); writing – review and editing (equal). **Daniela Calina:** Data curation (equal); investigation (equal); methodology (equal); supervision (equal); validation (equal); visualization (equal); writing – original draft (equal); writing – review and editing (equal).

## CONFLICT OF INTEREST STATEMENT

The authors wish to confirm that there are no known conflicts of interest associated with this publication and that there has been no significant financial support for this work that could have influenced its outcome.

## Data Availability

Not applicable.
